# From hostile worlds to multiple spheres: towards a normative pragmatics of justice for the Googlization of health

**DOI:** 10.1007/s11019-021-10006-7

**Published:** 2021-03-15

**Authors:** Tamar Sharon

**Affiliations:** Faculty of Philosophy, Theology and Religious Studies, Radboud University, PO Box 9103, 6500 HD Nijmegen, The Netherlands

**Keywords:** Digital health, Googlization of health, Commodification, Orders of worth, Spheres of justice, Datafication

## Abstract

The datafication and digitalization of health and medicine has engendered a proliferation of new collaborations between public health institutions and data corporations like Google, Apple, Microsoft and Amazon. Critical perspectives on these new partnerships tend to frame them as an instance of market transgressions by tech giants into the sphere of health and medicine, in line with a “hostile worlds” doctrine that upholds that the borders between market and non-market spheres should be carefully policed. This article seeks to outline the limitations of this common framing for critically understanding the phenomenon of the Googlization of health. In particular, the mobilization of a diversity of non-market value statements in the justification work carried out by actors involved in the Googlization of health indicates the co-presence of additional worlds or spheres in this context, which are not captured by the market vs. non-market dichotomy. It then advances an alternative framework, based on a multiple-sphere ontology that draws on Boltanski and Thevenot’s orders of worth and Michael Walzer’s theory of justice, which I call a normative pragmatics of justice. This framework addresses both the normative deficit in Boltanski and Thevenot’s work and provides an important emphasis on the empirical workings of justice. Finally, I discuss why this framework is better equipped to identify and to address the many risks raised by the Googlization of health and possibly other dimensions of the digitalization and datafication of society.

## Introduction

In recent years, the large consumer technology companies that have become the predominant architects of our digital environments have swiftly moved into the health and biomedical sector, positioning themselves as important facilitators of digital health and medicine. Apple’s ResearchKit software, for example, now allows medical researchers to carry out clinical studies using the iPhone and is currently being used by prominent medical institutions such as Yale, Stanford and Mount Sinai Hospital (Chan et al. 2018; Turakhia et al. 2019). Apple has also begun partnering with pharmaceutical companies who see potential in the iPhone and Apple Watch for virtual “at home” monitoring and trials. Alphabet, Google’s parent company, is exploring ways to make its data collection and data analysis expertise useful for health too. In 2014, Verily, Alphabet’s life science division, launched an ambitious project to “map human health” in collaboration with Duke and Stanford University. Verily is also partnering with European research institutes (Bloem et al. 2019) and working with pharmaceutical companies like Novartis and Pfizer on clinical trial development (Farr 2019), while other Alphabet subsidiaries, such as Google and DeepMind, are developing AI for medical applications, with some recent successes in the prediction of cardiovascular disease, eye disease, and breast and lung cancer. Amazon is also showing growing interest in the health domain. In addition to the acquisition of an online symptom checking and triage tools company in 2019 and the development of a machine learning tool for the processing of unstructured medical texts, it has also entered a partnership with the UK’s NHS, which allows its Alexa voice-assistant to offer NHS health advice to users at home. A number of these companies have also recently begun moving into the domains of electronic health record management, employee healthcare and health insurance. With the outbreak of the COVID-19 pandemic in early 2020, moreover, Alphabet, Facebook, Apple and Microsoft were quick to develop COVID-19 specific data collection tools, screening and testing facilities, digital contact-tracing infrastructure and other COVID-related research initiatives (see for example Kelion 2020 and Sharon 2020).

In line with the Googlization of earlier areas of human activity (Vaidhyanathan 2011),^1^ this *Googlization of health* (Sharon 2016) typically raises a series of apprehensions among critical scholars of digitalization and datafication. These include concerns about privacy and data protection, i.e. will these companies share personal data with advertisers, insurers or employers (Zuboff 2019)? Concerns about the use of personal or publicly generated health data for financial profit, i.e. how will these companies monetize their investments in the health domain (Powles and Hodson 2017, 2018)? Concerns about the new gatekeeping function these companies may have in accessing valuable health datasets, i.e. will the datasets they help generate be open to all (Boyd and Crawford 2012)? And concerns about the growing concentration of power of these private corporations in traditional public sectors like health and education (van Dijck et al. 2019; Prainsack 2020). These concerns are underpinned by an understanding that the Googlization of health, as a phenomenon that emerges at the intersection of digital capitalism and digital health, invokes an uncomfortable merging of what are often held to be by nature or by normative design separate spheres of human life—the market and health. The economic sociologist Viviana Zelizer (2011) has called this framing the “separate spheres”, or the “hostile worlds”, doctrine: the idea that the market and other spheres of social life are, and should remain, separate.

This article seeks to outline the limitations of this common framing for critically understanding the phenomenon of the Googlization of health. In particular, it argues that additional worlds or spheres are intersecting in this context, which signal a more comprehensive, multiple-sphere ontology, and the need for a broader analytical framework for identifying and addressing the many risks raised by the Googlization of health. In other words, while the hostile worlds doctrine sensitizes us to the risks caused by the intrusion of the market logic into the sphere of health and medicine—which is an undeniable aspect of the Googlization of health—it does not do enough to identify the risks caused by the intrusion of logics from *other* spheres, which are co-present in this phenomenon. The article proceeds as follows. The first part offers a brief overview of the hostile worlds doctrine as a long-established and prevalent framing in philosophy, social theory, ethics and more recently critical data studies. It then discusses an important shortcoming of this framing when applied to the Googlization of health. Namely, numerous non-market values are prevalent in the justification work carried out by corporate and other actors supporting the Googlization of health—including empowerment and equality, the democratization of knowledge, technical expertise and efficiency, health promotion and social good—a situation which is not properly captured by the market vs. non-market dichotomy of the hostile worlds framing. Rather than viewing these justifications as non-market value statements appropriated or co-opted for market aims, I suggest to view them as mobilizations of different “orders of worth” (Boltanski and Thévenot 2006; Sharon 2018). Not just the “market” and “civic” orders, but also the “industrial”, “project”, “vitalist” and other orders. In the second part, I discuss the added value of this perspective up and against the hostile worlds doctrine, as well as some of its normative limitations, which I address and improve on with Michael Walzer’s (1983) theory of justice. Finally, I show how this alternative, multi-sphere approach, what I call a “normative pragmatics of justice”, is better equipped to identify and address the many risks raised by the Googlization of health and possibly other dimensions of the digitalization and datafication of society.

## The hostile worlds doctrine and its limitations

### Separate spheres and hostile worlds: iterations in philosophy, (bio)ethics and critical data studies

The notion that social life is organized in distinctly different spheres, the market and the non-market sphere, which are each organized according to different and incommensurable principles, values and ends, has a long tradition in Western social theory and philosophy.^2^ It is also the theoretical architecture upon which public debate and cultural resistance to “putting a price” on things—from organs and embryos, to companionship, basic education, sex and waiting on line—takes place. As the philosopher Elizabeth Anderson (1990a, 1995) discusses in her account of the fundamental differences between these two spheres, while the mode of valuation in the market sphere is use or utility, the non-market sphere invokes modes of valuation including respect and appreciation. While the former is marked by impersonality or a freedom from personal ties and obligations, the latter is marked by an understanding of, and commitment to, others as part of a relationship. And while in the former goods are typically divisible (i.e. exclusive and rival), as is the case with commodities, in the latter they are typically shared: their worth lies at least in part in the fact that they are understood to be held in common.

This “separate spheres” or “hostile worlds” doctrine (Zelizer 2011) does not only posit that the market and non-market spheres are alien to each other, but that contact between them gives rise to moral contamination. That is, the application of market norms to goods that are best realized in the non-market sphere is not only seen as undermining the value of those goods, but as transforming them; corrupting and degrading them by treating them according to a lower mode of valuation than is appropriate to them (Anderson 1990a; Sandel 2012). The market is here posited as an ever-expanding and destructive force that displaces other, non-economic modes of valuation and relations (Walzer 1983; Habermas 1984, 1987; Radin 1996; Caliskan and Callon 2009; Satz 2010) and whose distinct normative reasoning risks seeping through every single dimension of human life (Foucault 2008; Brown 2015). Its borders, hostile worlds scholars argue, should thus be carefully managed—either by strengthening existing normative constraints that maintain certain goods outside of market relations, or by legal and institutional “blockages” (Walzer 1983). This defence of sphere separation has been particularly vehement in the domain of health and medicine (Anderson 1990b; Hoeyer 2007; Scheper-Hughes 2000; Sharp 2000; Satz 2010; Titmuss 1970), where human life, and by extension bodies and body parts, are held to lie beyond economic calculation, and where healthcare and medical research are seen as goods at risk of moral degradation should they succumb to the rationale of the market.

More recently, the hostile worlds doctrine has also become a commonly deployed framing in critical data studies, the growing body of scholarship that studies the impacts of digitalization and datafication processes on society and culture from a critical perspective. In this iteration of the hostile worlds doctrine, data sharing practices are framed in terms of either private benefit and financial profit *or* public benefit and social good. The notion of “digital labor” (Terranova 2000; Fuchs 2013), for example, has been paramount in this context. This term refers to the idea that the dominant model of capital accumulation of Internet corporations is based on the exploitation of users’ unpaid labor, who generate non-remunerated value for companies by creating content. On the one hand, scholars here denounce the commodification of data that are generated in non-market spheres; for example, while searching for information online, while using mobile applications on a personal device, or while communicating and sharing on social media platforms. On the other hand, they denounce the commodification of data that are generated for non-financial benefits, such as socializing, sharing information to help similar others, or contributing data for the improvement of public services or research. Sphere encroachment takes place in two ways in this context. First, when commercial entities appropriate personal data that flow freely in the public domain and transform them into profit (see for example Pasquale 2015; Zuboff 2019; van Dijck et al. 2019; Birch and Muniesa 2020). Second, and often as a response to this appropriation, when property rights are assigned to personal data so that they can be monetized, where monetization is proposed as a means of redressing the asymmetry between corporations and individuals generating data in the data economy (Lanier 2013; Lehtiniemi and Ruckenstein 2019).

Expanding on this reading of the commodification of digital data, in the context of digital *health* data, the hostile worlds doctrine has been used to highlight the commodification and exploitation for profit of, for example, patient experiences shared online (Lupton 2014), genomic data shared for research (Harris et al. 2013; Sterckx et al. 2013), and data generated by apps used by individuals to track and monitor health-related activities (Ajana 2017; van Dijck et al. 2019). Lupton writes, for example, “just as other forms of digital prosumption have been expropriated by capitalist enterprises in the interests of profit … so too patient experience prosumption has generated new avenues for commercial endeavours by enterprises that have seen the opportunity for expropriating value” (2014, p. 864). Van Dijck et al. (2019), in their recent book on the platformization of various sectors including health, speak of the “double promise” that platform operators intent on gathering personal health data employ: the promise of personalized solutions to medical challenges, but also the promise of public benefit and contributing to the common good of health research. Such critical studies often hone in on the discrepancy between the altruistic motivations of users sharing their health data and the commercial motivations of companies collecting data, where the latter’s mobilization of the lexicon of “sharing”, “doing good” and “philanthropy” facilitates a nonviolent intrusion of the market logic into a sphere of gift relations (Prainsack 2017). Here, much like Titmuss’ (1970) claims concerning blood donations, the commodification of health-related digital data is seen as having a corrosive effect on the norms of sharing and donating data for altruistic purposes.

Understood within this long tradition of hostile worlds, the Googlization of health seems like a flagrant instance of sphere contamination; of a novel encroachment, this time of the *digital* economy into the sphere of *digital* health and medicine. There are numerous benefits to applying this analytical framework to the Googlization of health. Namely, by situating the Googlization of health within the wider political economy of digitalization, this framing importantly calls attention to the potential deleterious effects of commodification of digital health that lie beyond privacy harms, such as harms to social justice, fairness and democratic control (Sharon 2018). That is, at a time when the regulatory and ethical vocabulary for addressing the negative impacts of digitalization is predominantly privacy and consent, the hostile worlds framing contributes to broadening this discussion to more collective values. However, and while this is an important shift, it is questionable how well the hostile worlds doctrine applies to the Googlization of health. In the following section I discuss a significant limitation posed by the Googlization of health to the hostile worlds doctrine, by focussing on the sphere-specific value qualification within the hostile worlds framing and comparing this to the moral lexicon used to justify the Googlization of health.

### Sphere-specific value repertoires

The dichotomy erected by the hostile worlds doctrine between the market and the rest, in addition to allocating specific goods, norms and practices to either spheres, also allocates moral values in this way. The tendency here is to situate most moral values, particularly those relating to the common good, within the non-market sphere, with the exception of two values which are seen as characteristic of the market: freedom and utility. Thus, the market is seen (both by proponents and hostile world critics), as a sphere where individuals are (purportedly) free, within the limits of the law, to pursue their own interests; to buy and sell what they please, be these apples or kidneys, cleaning services or sex. This is the libertarian moral argument in favor of markets (Nozick 1974). In addition, the market is understood as a sphere where people are enabled to make mutually advantageous trades, and where successful commercial transactions can bring benefits to all parties involved, thereby increasing general welfare, or social utility. This is the utilitarian argument in favor of markets, according to which, as Adam Smith (1776) posited, the pursuit of self-interests in the market place frequently promotes, even if unintentionally, the interests of society.

While allowing for a certain morality of the market, albeit a limited one embodied in these values of market freedom and utility, theorists who ascribe to the hostile worlds framing tend to be very critical of it. For example, against libertarian arguments for markets, they question if market choices are always made freely or if some kind of coercion is exercised. Thus, people may be acting out of necessity when ceding to the sale of blood, body parts or other goods (Satz 2010; Sandel 2012). Against utilitarian arguments for markets, on the other hand, defenders of sphere separation maintain that the very attempt to weigh preferences without judging them, to measure all goods on a single scale, is flawed, because goods differ substantively in kind (Sandel 2012). It is precisely the attempt to valuate some goods like economic ones that depreciates and corrupts them in this view.

The hostile worlds doctrine thus offers both a critique of the market in terms of its moral deficiency (most moral values are situated in the non-market sphere), as well as a compelling critique of those limited moral values which are held to be specific to the market. But does this critique and value qualification of spheres hold up in the case of the Googlization of health? One way of attempting to answer this is to take a close look at the value statements that are made by actors to justify the recent and increasing involvement of tech and data corporations in the field of health and medicine, or to carry out a “justification analysis” of such statements, an approach for studying moral evaluations made in public debates, statements, and text-based materials (Lehtonen and Liukko 2010; Ylä-Anttila and Luhtakallio 2016; Sharon 2018). Such a justification analysis, even a cursory one, indicates that statements made by corporate actors to justify their involvement in health and medicine do not typically mobilize market values (freedom and utility), but non-market values. These values include *empowerment and equality*—understood in terms of a democratization of medical knowledge for patients; *data expertise and efficiency*—understood here not as market efficiency (the matching of preferences of two parties in a market exchange), but as the streamlining of the onslaught of health data the medical community needs to handle; and *social value*—which these corporations claim to contribute by addressing some of society’s most pressing challenges, including healthcare. Importantly, and as will be further discussed below, this is not to say that these justification statements should be taken at face value as either the true motivations of these actors or as a description of what actions are being carried out in this context. Rather, these indicate a discrepancy between the sphere-specific value repertoires identified in the hostile worlds framing and those mobilized in the phenomenon of the Googlization.

### Examples of the presence of non-market values in the justifications for the Googlization of health^3^

Empowerment and equality are common values that are drawn upon by corporate actors in this context. For example, in an interview on Apple’s health strategy, the company’s Vice President of Health, Dr. Sumbul Desai, explains that “democratizing data” is one of the company’s main aims. Discussing the recent ECG function added to the Apple Watch in 2019, she says, “we think that empowering a customer and really democratizing the information for a customer to use (…) so that they can engage in their health more effectively is what we’re trying to do (...) By putting the ECG in someone’s hand (...) it’s really a way for you to engage with your physician in a different way” (in Comstock 2019). Tim Cook, Apple’s CEO, has also emphasized the democratization of medical data as one of Apple’s aims: “We are democratizing it [data]. We are taking what has been with the institution and empowering the individual to manage their health” (Cook 2019). While this appeal to democratization and empowerment may be understood as an invocation of the value of freedom, what is being invoked here is not the market value of freedom as “exit”, but the political value of freedom as “voice” (Hirschman 1970).

In addition to empowering patients and citizens, the data expertise of these companies is advanced as an important contribution in an age of digital and data-driven health and medicine. If Google’s mission statement has always been to “organize the world's information and make it universally accessible and useful”, Verily, Alphabet’s life sciences subsidiary, has a similar, data-driven health-related mission, to “make health information useful so people can live healthier lives”. Indeed, according to Verily’s chief medical officer, Jessica Mega, the one thread that unites the company’s various health-related projects is information. As clinicians, patients, and providers of health services are confronted with exponentially growing datasets, she explains that Verily’s value lies in offering the tools to manage and streamline these data: “What we’re trying to do […] is to stay ahead of the infrastructure you need to handle this next wave of data” (in Brodwin 2019). Similarly, in a blog post announcing Verily’s recent partnership to launch an opioid addiction treatment center in the US, Verily’s contribution is framed in terms of “addressing the critical information gap” (Verily 2019) that exists in addiction medicine, by tapping into their capabilities and expertise in building health data platforms. Providing more efficient ways of gathering, managing and analyzing data is touted by most of these companies as their direct contribution to health and medicine, from providing the “essential capabilities necessary to help clients drive their digital transformations: deep industry expertise, data and analytics, and actionable insights” (IBM Watson Health n.d.), to using technology like an iPhone to increase patient enrollment and thus increase data collection (Apple n.d.).

An explicit and recurrent principle in the justification statements found here is the generation of social value and contributing to the common good, by addressing society’s most complex challenges—including healthcare. This differs from more traditional forms of philanthropy, by which wealthy CEOs donate parts of their fortunes to social causes including research into various diseases and public health projects. These types of philanthropic efforts are *also* being undertaken by leaders at tech corporations, for example in the Bill and Melinda Gates Foundation or the more recent Chan Zuckerberg Initiative, which has pledged to spend $3 billion over the next decade to support scientific research (Love 2016). But a number of key initiatives in the Googlization of health diverge from this traditional understanding of philanthropy on two dimensions. First, creating social value is presented as the main aim and expected general impact of what these companies do.^4^ And second, this contribution is channeled specifically through the technical expertise that these companies provide—not their financial resources.

Demis Hassabis, for example, founder of DeepMind, Google’s AI offshoot, discusses how technology will likely be more successful than humans at advancing societal good:

If you look at the challenges that confront society today: climate change, sustainability, mass inequality—which is getting worse—diseases and healthcare, we’re not making progress anywhere near fast enough in any of these areas. Either we need an exponential improvement in human behavior (…) or we need an exponential improvement in technology. If you look at current geopolitics, I don’t think we’re going to be getting an exponential improvement in human behavior any time soon. That’s why we need a quantum leap in technology like AI. (in Heath 2018)

For Tim Cook, Apple’s uniqueness lies in the company’s clear motivation to “change the world” and to “be a force of good”, by focusing on “what will help people, rather than what will make the company money” (in Lashinsky 2017). This holds for the company’s recent interest in health as well. Cook explains that, while health is certainly a lucrative market, many of Apple’s health-related initiatives are not profit-driven. Speaking of the ResearchKit software for example, he says: “There’s no business model there. Honestly, we don’t make any money on that. But it was something that we thought would be good for society and so we did it” (in Lashinsky 2017). Indeed, in an interview given a couple of years later, Cook went as far as to claim that he expects Apple’s “greatest contribution to mankind” to be in health (Cook 2019).

Other values that have been identified in statements and promotional material of these companies include *innovation* and *trouble-shooting*—that is seen as being brought to bear from the outside on a convoluted, lethargic and overly bureaucratic healthcare and medical system; *inclusivity* and *diversity*—by allowing more individuals to participate in medical research via consumer technology devices; and *solidarity*—enabled by the platforms that promote data sharing between citizens and patients and medical researchers (Sharon 2018).

Framed within the hostile worlds doctrine, such a mobilization of non-market values by market actors is typically understood as a rhetorical ploy, used to lure unsuspecting individuals to donate their health data for what is presented as societal good but actually aims to enhance corporate profit. It is precisely this deceptive and exploitative feature which is captured by the notion of “free digital labour” referred to above. To be sure, instrumental uses of non-market values for market aims are a reality in the age of digital capitalism, as are some forms of digital labour. Furthermore, promotional materials and interviews do not, of course, communicate actual decision-making processes, nor do they provide access to the authentic or real motivations of actors. The difference between what is said and what is done can be vast.

However, there are several reasons to push back against this type of explanation for the presence of non-market justifications for the Googlization of health as too quick. First—and while it is questionable if the “real” motivations of actors are *ever* accessible to observers—these materials do convey something about the scope of possibilities for action, especially in relation to concrete technology design. In this sense they may certainly contribute to the shaping and guiding of decision-making processes and technological futures, making them to some extent performative (Austin 1962; Foucault 1970; Latour and Woolgar 1979; Verbeek 2005). Moreover, as I shall outline below, mapping out these different moral justifications can be very helpful for identifying which decision-making processes and actions are to come, and what risks they entail. Finally, value statements drawing on non-market moralities in order to justify the involvement of data corporations in health and medicine are also common among *non-*corporate actors in this context. For example, the technical aptitude of these companies is often invoked by medical scientists. Thus, in the study protocol of the Personalized Parkinson Project, a partnership between Verily and Radboud University Medical Center in the Netherlands, one reads that Verily’s “analytics capabilities” are expected to help researchers to “address research questions of great scientific and clinical value” (Bloem et al. 2019, p. 8). In relation to the Apple ResearchKit, a Duke University scientist states that “[w]e’ve gone as far as we can with traditional research. Now we have technology in our pockets that lets us go even further” (Apple n.d.). In addition, the value of the creative and innovative approach these companies bring to healthcare is also echoed in statements made by medical professionals, policy makers and hospital managers. Speaking of Amazon’s recent interest in geriatric health, for example, one physician claims, “Health care—especially for seniors—is at its breaking point and is ripe for disruption (…) what Amazon has figured out is that to provide high-quality health care for seniors, physicians must be innovative” (Schayes 2018). Similarly, claims to patient empowerment and participation, as a result of these corporations seeking to “meet customers where they are” (Brodwin 2020), and seeking to develop design features that enable altruistic data sharing with researchers (Wilbanks and Friend 2016), have also been identified by medical scientists, tech analysts and non-profit scientific organizations commenting on these developments.

In other words, the justifications that warrant the involvement of these corporations in the health and medical sphere are not limited to justifications drawn from the market sphere, and this points to a discrepancy between the sphere-specific value qualification of the hostile worlds framing and the moral lexicon of the Googlization of health. Rather than explaining the presence of these non-market moral orientations at work in the Googlization of health by pigeonholing them solely as statements that have been appropriated for market aims, a framework that moves beyond the hostile worlds doctrine is needed.

## Towards a multi-sphere ontology and a normative pragmatics of justice

### Orders of worth and the plurality of common goods

A fruitful way forward here is to view these value statements as representative of what the French sociologists Luc Boltanski and Laurent Thévenot have called “orders of worth” in their seminal work *On Justification* (2006). Orders of worth are coherent vocabularies of argumentation and justification that are organized around different visions of the common good and of what is just. They are general grammars of social order or political bonds oriented toward justice, which people appeal to when in situations of (non-violent) conflict. That is, when they need to justify their positions and actions, or when they are criticizing the positions of others. For each order, one fundamental or “higher common principle” helps demarcate that which is valuable or good from that which is of lesser worth.

Thus, statements that emphasize the data management expertise that these companies provide to the health and medical sector as something valuable and worthy draw on what Boltanski and Thévenot call an “industrial” order of worth. The industrial order distinguishes between functionality and professionalism as valuable and chaos and unproductivity as worthless. Conversely, statements that emphasize the democratization of medical information, just as statements that emphasize doing good for society, draw on what the authors call a “civic” order. In the civic order, the common good is conceptualized in terms of social value and collective benefit and values like solidarity, equality and participation are foregrounded. Statements that emphasize “thinking out of the box”, “disruption” and a novel approach to health and medicine that these companies bring from the outside draw on a “project” order (Boltanski and Chiapello 2005). Here the common good is conceptualized in terms of innovation, and emphasized values include experimentation and activity as opposed to stagnation and prudence. In *On Justification*, Boltanski and Thévenot identified six orders of worth based on their analysis of contemporary French corporations, to which two additional orders have been supplemented in further work, for a typology of eight ideal types of justification. In addition to those mentioned, also a “market” (enhanced wealth creation and utility), a “domestic” (familial ties and tradition), an “inspired” (creativity and uniqueness), “fame” (public recognition), and a “green” (ecology) order (Moody et al. 2000).

This typology is characterized by both a radical pluralism—there is a diversity of ways of specifying the common good—and what we might call “non-relativism”—this radical pluralism does not result in moral relativism.^5^ In terms of the latter, moral relativism is averted in this framework because there is not an infinite number of orders of worth. Indeed, insofar as these orders are brought to work in situations where people are attempting to justify themselves, that is, to persuade others of the goodness of their actions, these orders need to be recognizable to others. “When one is attending to the unfolding of disputes,” they write, “one sees that they are limited neither to a direct expression of interests nor to an anarchic endless confrontation between heterogeneous worldviews clashing in a dialogue of the deaf” (2006, p. 13). This prerequisite of recognizability means that orders of worth only make sense in light of some *shared* understanding of justice, which in turn implies that orders of worth are historical and contingent—specific for a particular society at a given time. Orders of worth can thus vanish and they can emerge. The “project” and the “green” orders that Boltanski, Thévenot and colleagues identified in subsequent works, for example, have been identified as emergent orders. Similarly, in the justification analysis of the Googlization of health that I carried out (Sharon 2018), I identified the order of “vitality”, organized around the value of health as a higher common principle, as an emergent and frequently mobilized order of worth in this context.^6^

More than the avoidance of moral relativism however, it is the first feature of this typology which is of particular interest here: the radical plurality of orders of worth. Boltanski and Thévenot’s main line of reasoning is that societies are made up of an interweaving of multiple understandings of justice and forms of agreement. This pluralism allows us to move beyond the hostile worlds doctrine in several significant ways, and thus offers fertile ground for a framework that would be better equipped to address the risks involved in a phenomenon like the Googlization of health. First, where the hostile worlds doctrine applied to the Googlization of health sees only the market and the non-market spheres, the framework of orders of worth allows for a much more complex moral landscape, where multiple orders are co-present. This framework can thus account for a broad diversity of value orientations that seem to be at work in a phenomenon like the Googlization of health.^7^ Furthermore, where the hostile worlds doctrine situates the common good primarily in what Boltanski and Thévenot would call the “civic” order of worth, which foregrounds solidarity and inclusion, in Boltanski and Thévenot’s framework the civic order does not have a monopoly over the common good and other moral values. Rather, each and every order of worth is grounded in some conception of the common good; each one advances a model of agreement and justice. In other words, even the market order—whose pool of available values is limited at best to the values of market freedom and utility in the hostile worlds framing—qualifies as an order which provides a rich and meaningful sense of justice grounded in a conception of the common good and provides a moral orientation to action. It too requires that persons, in order to be considered worthy, sacrifice individual interests for a collective. They write, “moral capacity is presupposed in the construction of an order of market exchanges among persons, who must be capable of distancing themselves from their own particularities in order to reach agreement about external goods that are enumerated and defined in general terms” (2006, p. 27). In other words, in comparison to the hostile worlds framing, this framework offers both additional orders, as well as additional conceptualizations of the common good.

### Addressing Boltanski and Thévenot’s normative deficit

The orders of worth framework, however, is a descriptive one. While it is useful for identifying numerous orders of worth that may simultaneously be at work, it does not offer any critical evaluation of these different orders (all common goods are equally worthy), and it does not provide tools for making normative claims about the mobilization of one or another order in different situations. To resolve this normative deficit, Michael Walzer’s theory of justice can be useful.

In *Spheres of Justice* (1983), Walzer develops a theory of justice based on the autonomy of spheres within a multiple sphere ontology. Social life, according to Walzer, is organized into different spheres—the market, politics, welfare, education, etc.—in which different social goods are distributed according to different principles of justice. A just society, Walzer maintains, is one where advantage in one sphere cannot be converted into advantage in another. Wealth, for example, an advantage procured in the market sphere, should not translate into better education, better medical care or political influence. Just as having political power, for example holding office, should not translate into benefits in these other spheres. Such illegitimate conversions, or transgressions between spheres, as many hostile worlds theorists would also argue, can lead to a loss of meaning of those goods which succumb to the distributive logic of the wrong sphere. An additional, and perhaps greater risk for Walzer, thinking in terms of a multiple and not just a dual sphere ontology, is the accrual of advantage or power across all spheres, a situation in which inequalities align across spheres and which Walzer calls “tyranny”. Walzer’s theory of justice thus provides a theory of justice by which to critically evaluate the conversions or intrusions in a multiple sphere setting, which the orders of worth framework does not.

Bringing Walzer into conversation with Boltanski and Thévenot is not coincidental. The French sociologists acknowledge that Walzer’s *Spheres* *of Justice* was an important inspiration for their work on the orders of worth. They write, “This pluralism [of orders] brings our position close to the one developed by Michael Walzer and, as it did for Walzer (…) it led to our interest in a theory of justice that would take into account the diversity of ways to specify a common good” (2006, p. 14). And both Walzer and Boltanski and Thévenot acknowledge one of seventeenth century philosopher Pascal’s (1966) *pensées* as an earlier source of inspiration for the idea that there are different orders or logics, and that the attempt to dominate regardless of these differences is a form of “tyranny”. Still, the two frameworks differ importantly, a point it is useful to linger on in order to understand the added value that each of these frameworks can bring to the study of a phenomenon like the Googlization of health.

First of all, orders of worth are not the same as “spheres”, in the sense that either Walzer or hostile worlds framings appeal to different spheres. Orders are more like repertoires or discourses—articulations of practices and values that give order to, or that are specific to, a sphere. Thus, the civic order can be understood as an articulation of the practices of the sphere of democratic politics, the domestic order an articulation of the practices of the sphere of family relations, etc. In this sense, the same sphere may be organized along the requirements of different orders. Family life and households (the “sphere of the family”) may be run “civically” (like democracies, with one vote per family member), or “industrially” (like an efficient factory), or more “domestically” (like a patriarchy); just as factories may be run civically, industrially or domestically, etc.^8^

Secondly, and related to this, the orders of worth framework can be seen to some extent as a critique of Walzer’s theory of justice: Boltanksi and Thévenot were critical of the abstract and universalizing tendencies of moral philosophy. Indeed, Boltanski and Thévenot developed their framework as both a critique of, and a means of bridging, moral or political philosophy and sociology. For them, while moral philosophy focuses too much on abstract principles of justice, typically universalizing ones, sociology—at least the school of Bourdieusian, critical sociology that dominated in French scholarship at the time they were writing—fails to take seriously individuals’ sense of justice.^9^ Their framework seeks to address each of these lacunae. First, by shifting from discussing abstract principles of justice to examining empirical situations of agreement and disagreement. Secondly, by following the arguments and criticisms of actors without reducing these to power and ideology. This, they argue, is a means of investigating the “*situated* sense of the just” (Boltanski and Thévenot 2000, p. 216, emphasis added).

This emphasis on what we may call the pragmatics of justification, can allow for a more precise and heedful attention to how orders of worth travel between spheres: while Walzer’s spheres can be understood as institutions, orders of worth are more ambulatory, flexible (two or more orders are often combined in a justification), and adaptable to specific situations. Coupled with the normative evaluation of sphere transgressions provided by Walzer’s theory of justice, this pragmatics of justification—or now a *normative pragmatics of justice*—may allow us to better identify various “micro” transgressions and illegitimate importations and conversions of orders of worth into spheres. And not just from the market sphere into the sphere of health and medicine, but from all other spheres as well.

### A new line of inquiry

Such a normative pragmatics of justice can open up a whole new line of critical inquiry. It enables the formulation of questions such as: which sphere transgressions and importations of orders of worth are detrimental, and which ones can be beneficial and lead to ethical innovation? Which ones contradict the traditional values of a sphere and why and where is this problematic? Which ones are solely instrumental to realizing the values of a sphere, and which ones risk crowding those values out?

Novel risks, which may be specific to the phenomenon of the Googlization of health but may also be implied in the broader process of the digitalization of health and of society, can then be identified and described. For example, we can begin to ask, “what happens to the sphere of health and medicine when the civic order of worth becomes dominant or hegemonic?” The civic order of worth, with its appealing emphasis on inclusivity, rights, reciprocity and democracy, is a very popular one in contemporary liberal societies, dear to both liberal enthusiasts and critical scholars. In the context of health and medicine, a rise to prominence of the civic order of worth has been identified by many scholars in the form of the so-called “participatory turn”, new emphases on patient autonomy and empowerment, and more equal relationships between experts and patients (Eysenbach 2008; Prainsack 2014; Topol 2015). Above, we have also seen that data corporations moving into health and medical research also frequently mobilize the civic order of worth to justify this involvement. But in the sphere of health and medicine, this rise of the civic order of worth has developed alongside a devaluation of the domestic order of worth, typically associated with paternalism, unequal relationships between doctors and patients and the exploitation of research participants. While this devaluation of the domestic in favour of the civic brings with it important ethical benefits, it will also come at a price, insofar as the domestic order of worth also foregrounds the values of caring, watching over vulnerable others, and trust. For example, how will the prominence of the civic affect the focal practice of medicine as the provision of care (Mol 2008)? How will clinicians and researchers fulfil the new obligations of “giving back” that reciprocal, civic relationships with their patients and research participants entail? And in terms of the value of personal data for medical research and care, to what extent will a civic order of worth transform the sharing of personal data into a moral duty, for example in new calls for “data solidarity” (Rathenau 2020)?

Conversely, what happens when a vitalist order of worth, that views health as the higher common principle, becomes dominant in the sphere of health and medicine? To a large extent, the vitalist order, in combination with the domestic, has always been the dominant order of worth of the sphere of health and medicine. But the prominence of this order can also legitimize practices that risk undermining values from other orders. Ubiquitous, constant health (self-)monitoring via mobile apps, virtual medical assistants and health maps, for example, may well lead to more preventive and personalized medicine and better health outcomes, as proponents uphold (Steinhubl et al. 2013; Topol 2019). But it also curtails individual autonomy and privacy. The prominence of the vitalist order may also legitimize the presence of data corporations and their contributions to healthcare and medical research while downplaying the various costs of this involvement—in terms of the market price the public sector may need to pay for the treatments and services that these corporations will develop, or in terms of a loss of democratic control over health data as a public resource.

What risks, moreover, does the industrial order, which frames health and medicine as problems of data management, pose to the sphere of health and medicine? Does this lead to a redefinition of who counts as a medical expert? How does this influence how medical knowledge will be produced in the future? How will this reshape the work routines of healthcare professionals and scientists? And how will this determine who has a say in shaping research agendas? When the industrial order becomes dominant in the sphere of health and medicine, the expertise of data corporations moves from being a purely technical expertise to an expertise in health and medicine, that legitimizes their initiation of research projects, possibly at the cost of research into diseases that they find less valuable. Alphabet, for example, has recently become involved in Parkinson’s research at several levels, in the form of 23andMe’s Parkinson’s Genetics Initiative and the Personalized Parkinson’s Project mentioned earlier. This particular interest in Parkinson’s research has coincided, as has been openly spoken about, with Sergey Brin’s discovery that he carries a form of hereditary Parkinson’s disease (Goetz 2010).

The importation and rise to dominance of any order of worth will present risks, in the form of trade-offs and the undermining of values prominent in competing orders of worth. These need to be carefully scrutinized and weighed. But more than any other type of encroachment, it is perhaps the importation and growing prominence of a specific type of industrial worth into the sphere of health and medicine—in the form of expertise in data collection, analysis and infrastructure—which presents the most dangerous conversion we are witnessing today. It is by means of their technical expertise that data corporations are becoming necessary passage points in health and medicine, and all other sectors of society that are undergoing digitalization. A recent example of this can be seen in the contribution that Apple and Google made to the global COVID-19 pandemic response in April 2020, by developing a platform for automating and running digital contact-tracing apps. As a platform that became the API that almost all countries developing contact-tracing apps in the end adopted—even when the platform’s decentralized protocol conflicted with the initial preferences of some countries to have centralized systems—Apple and Google succeeded in de facto shaping how contact-tracing would be carried out in the digital age, and thus determining an important component of global public health policy. In this instance, their technical expertise, or their industrial worth, to use the framework expanded in this paper, enabled them to encroach into the spheres of both public health and global politics (Sharon 2020).

## Conclusion

In this article I have argued that the hostile worlds doctrine, an age-old framing prevalent in social theory, political philosophy, (bio)ethics and more recently critical data studies, has limited value in the context of the Googlization of health. This is not to say that it has no value: it is helpful in broadening critical perspectives on this phenomenon beyond the narrow lens of privacy harms, insofar as it situates this phenomenon within the political economy of data and the commodification of health data. But it is insufficient for identifying risks of conversion from other non-market spheres and possible new forms of what Walzer called “tyranny” that the Googlization of health introduces. A manifestation of this limitation can be found in the discrepancy I highlighted between the sphere-specific value qualification of the hostile worlds framing and the moral lexicon found in public justifications legitimizing the Googlization of health. Here, value statements made by both corporate and non-corporate actors involved in the Googlization of health consistently draw on values from numerous non-market spheres. Rather than dismiss these statements as deceptive or co-opted, I suggested that this justification work is an indication of the co-presence of multiple spheres and the need for an analytical framework that can account for this multiplicity.

Boltanski and Thévenot’s framework of orders of worth, which assumes a plurality of orders each organized around a different conception of the common good, provides a useful alternative. Here multiple orders of worth, in addition to the “market” and the “civic” order, interweave within any given society and each of these, including the market order, are characterized by different moral orientations towards the common good. There is a certain normative deficit in the orders of worth framework, however, which I suggested can be resolved by drawing on Walzer’s theory of justice as the autonomy of spheres in a multi-sphere ontology. Combined, these frameworks provide what I called a *normative pragmatics of justice*, that bring together both a keen, empirical attention to travelling orders of worth and their deployment in different spheres, and a critical and normative evaluation of importations and conversions of orders of worth. This framework opens up a new line of inquiry into the Googlization of health and allows us to identify many risks that the hostile worlds framing, with its focus on market encroachments, remains blind to, in the form of civic, industrial, vitalist or other importations and conversions into the sphere of health and medicine.

Importantly, both Boltanski and Thévenot’s and Walzer’s frameworks imply a historical contingency that is valuable in the case of a novel phenomenon like the Googlization of health. These ontologies indicate a dynamic interaction of orders and spheres, where different orders and spheres may be dominant in different historical periods. While Boltanski and Thévenot speak of the disappearance and emergence of new orders of worth, Walzer understands the history of liberalism as the erection of boundaries between spheres, and in particular the placing of proper limits on the political sphere, to limit the reach of government. If this is liberalism’s greatest achievement, according to Walzer, at the time he developed his theory of justice he had become concerned with growing transgressions from the market sphere, to which liberalism seemed to him less sensitive. “We need to look closely,” he warns, “at the ways in which wealth, once political tyranny is abolished, itself takes on tyrannical forms” (1984, p. 321). The lines between spheres, in other words, need to be constantly redrawn. Similarly, with the Googlization of health and other sectors of society, including education, news provision, and city planning, we may be facing new transgressions from existing known spheres as well as transgressions from new and emergent spheres that a focus on commodification and commercialization do not do enough to sensitize us to. As I have argued, we should in particular be wary of a new industrial tyranny, by which technical expertise, especially in the form of data expertise, translates into an asset worthy of conferring power in non-industrial spheres, such as the sphere of health and medicine, but also the sphere of politics. The challenge, in what may be a new historical configuration of spheres largely being redefined by the processes of digitalization and datafication, is to redraw the lines and define new limits of convertibility in ways that secure justice in our contemporary moment.

## Data Availability

Not applicable.

## Footnotes

1 In his book *The Googlization of Everything* (2011), Vaidhyanathan identifies the “Googlization” of knowledge (books), memory, the world (the global sphere), and “us” (universal surveillance), though not the area of health, which in 2011 was still unchartered territory for Google. Drawing on Vaidhyanathan’s term to denote the expansion of Google’s reach into ever more areas of social life, I also use it more broadly, to include the efforts of other tech corporations, such as Apple, Amazon, Facebook and Microsoft, whose initial interests also were not directed towards the health sector.

2 Hirschman (1982), for example, traces this notion back to the nineteenth and early twentieth centuries, in critics of capitalism, both Marxist and conservative.

3 A number of the examples presented here are taken from the broader justification analysis carried out in Sharon (2018). The material used there included interviews, conducted by myself or made public, with stakeholders involved in the Googlization of health (such as industry representatives, scientists at public research institutions partnering with or open to partner with tech corporations, patients and policy makers), promotional material, and policy reports on digital health, AI, and public–private partnerships.

4 This first dimension resembles what has been coined “philanthrocapitalism”, the conflation of business aims with charitable efforts (Economist 2006).

5 This limited pluralism, or pluralism without relativism, gives rise to some tension of course. Namely, the question of how plurality is possible when legitimacy seems to require universality (2006, p. 39). This tension is resolved in the “polity model” that the authors develop in *On Justification*, a set of axioms or binding conditions which any order of worth must satisfy in order to be constituted as such.

6 The order of vitality is particularly interesting in the context of the COVID-19 pandemic outbreak which took place during the time of writing, and during which public health became a common justification for using technology to address the pandemic, often up against civic justifications around privacy and other civil rights. See for example the justification analysis conducted by Siffels (2020) of the public debate on the development of contact-tracing apps.

7 This goes further than some recent scholarship that suggests that we are witnessing a merging of social and financial profits in contemporary capitalism in ways that the hostile worlds doctrine fundamentally misses (see for example McGoey 2015 and Prainsack 2017). This scholarship is helpful in moving beyond the strict separation at the heart of the hostile worlds framing, but still falls short of identifying multiple orders and spheres. Prainsack, for example, argues that current health entrepreneurism is the result of new convergences between social and financial value—but only of these two modes of valuation. I argue that beyond these new convergences of two types of value, additional ones are also at stake, complicating a phenomenon like the Googlization of health even more.

8 I thank Andrew Hoffman for help in elucidating this point.

9 Boltanski developed his “sociology of critique” in response to Bourdieusian “critical sociology”, which he saw as being based on trying to unveil the “real” motivations and desires of people and explaining them in terms of habitus and structure, with the result of developing a problematic asymmetry between the critical researcher and actors who were presented as being steeped in illusion (Boltanski et al. 2014). Instead, the sociology of critique implies engaging seriously, and symmetrically, with different justifications. Incidentally, this is in line with the plea made earlier not to dismiss the non-market value statements made in the context of the Googlization of health as rhetorical and inauthentic from the outset.

## References

[CR2] Ajana B (2017). Digital health and the biopolitics of the Quantified Self. Digital Health.

[CR3] Anderson E (1990). The ethical limitations of the market. Economics & Philosophy.

[CR4] Anderson E (1990). Is women’s labor a commodity?. Philosophy & Public Affairs.

[CR5] Anderson E (1995). Values in Ethics and Economics.

[CR9] Apple, n.d. https://www.apple.com/lae/researchkit/. Accessed 19 December 2020.

[CR10] Austin J (1962). How To Do Things With Words.

[CR11] Birch K, Muniesa F (2020). Assetization: Turning Things into Assets in Technoscientific Capitalism.

[CR12] Bloem B, Marks W, Silva de Lima A (2019). The Personalized Parkinson Project: Examining disease progression through broad biomarkers in early Parkinson’s disease. BMC Neurology.

[CR14] Boltanski L, Thévenot L (2000). The reality of moral expectations: A sociology of situated judgement. Philosophical Explorations.

[CR13] Boltanski L, Chiapello E (2005). The New Spirit of Capitalism.

[CR15] Boltanski, L., and L. Thévenot. 2006. *On Justification: Economies of Worth*. Princeton: Princeton University Press.

[CR86] Boltanski, L., A. Honneth, and R. Celikates. 2014. Sociology of critique or critical theory? Luc Boltanski and Axel Honneth in conversation with Robin Celikates. In *The Spirit of Luc Boltanski: Essays on the ‘Pragmatic Sociology of Critique’*, ed. S. Susen and B.S. Turner, 561–589. London: Anthem Press.

[CR16] Boyd D, Crawford K (2012). Critical questions for Big Data. Information Communication & Society.

[CR17] Brodwin, E. 2019. We talked to the top scientist at Alphabet’s life-sciences company about the common thread uniting all its seemingly random health projects—And how she plans to spend $1 billion. *STAT*. https://www.businessinsider.com/verily-google-alphabet-ceo-shares-common-theme-behind-projects-2019-1?international=true&r=US&IR=T. Accessed 13 April 2020.

[CR18] Brodwin, E. 2020. How Amazon Pharmacy could ramp up pressure on the prescription drug industry. *STAT*. https://www.statnews.com/2020/11/18/amazon-pharmacy-pbms-walmart-optum/?utm_source=STATNewsletters&utm_campaign=b3a0207d21-health_tech_COPY_01&utm_medium=email&utm_term=0_8cab1d7961-b3a0207d21-151653869. Accessed 19 December 2020.

[CR19] Brown W (2015). Undoing the Demos: Neoliberalism’s Stealth Revolution.

[CR20] Caliskan K, Callon M (2009). Economization, part 1: Shifting attention from the economy towards processes of economization. Economy & Society.

[CR21] Chan Y, Bot B, Zweig M (2018). The asthma mobile health study, smartphone data collection using ResearchKit. Scientific Data.

[CR22] Comstock, J. 2019. Apple’s health strategy: Democratizing health information. https://www.mobihealthnews.com/content/apple’s-health-strategy-democratizing-health-information. Accessed 13 April 2020.

[CR23] Cook, T. 2019. Interview with J. Cramer. https://www.cnbc.com/2019/01/08/apple-ceo-tim-cook-interview-cnbc-jim-cramer-transcript.html?__source=twitter%7Cmain. Accessed 13 April 2020.

[CR25] Eysenbach G (2008). Medicine 2.0: Social networking, collaboration, participation, apomediation, and openness. Journal of Medical Internet Research.

[CR26] Farr, C. 2019. Google sister-company Verily is teaming with big pharma on clinical trials. *CNBC.* https://www.cnbc.com/2019/05/20/alphabet-verily-doing-clinical-trials-with-novartis-sanofi-pfizer.html. Accessed 11 April 2020.

[CR27] Foucault M (1970). The Order of Things.

[CR28] Foucault, M. 2008. *The Birth of Biopolitics: Lectures at the Collège de France 1978–1979.* Trans: Graham Burchell. New York: Palgrave.

[CR29] Fuchs C (2013). Theorising and analysing digital labour. The Political Economy of Communication.

[CR30] Goetz, T. 2010. Sergey Brin’s Search for a Parkinson’s Cure. *Wired.* https://www.wired.com/2010/06/ff-sergeys-search/. Accessed 13 April 2020.

[CR31] Habermas J (1984). The Theory of Communicative Action, Volume 1, Reason and the Rationalization of Society.

[CR32] Habermas J (1987). The Theory of Communicative Action, Volume 2, System and Lifeworld: A Critique of Functionalist Reason.

[CR33] Harris A, Wyatt S, Kelly S (2013). The gift of spit (and the obligation to return it). Information, Communication and Society.

[CR34] Heath, N. 2018. Google DeepMind founder Demis Hassabis: Three truths about AI. *TechRepublic.* https://www.techrepublic.com/article/google-deepmind-founder-demis-hassabis-three-truths-about-ai/. Accessed 13 April 2020.

[CR35] Hirschman A (1970). Exit, Voice, and Loyalty: Responses to Declines in Firm, Organizations and States.

[CR36] Hirschman A (1982). Rival interpretations of market society: Civilizing, destructive, or feeble?. Journal of Economic Literature.

[CR37] Hoeyer K (2007). Person, patent and property: A critique of the commodification hypothesis. BioSocieties.

[CR38] IBM Watson Health. n.d. https://www.ibm.com/watson-health/about. Accessed 13 April 2020.

[CR39] Kelion, L. 2020. Coronavirus: NHS turns to big tech to tackle Covid-19 hot spots. https://www.bbc.com/news/technology-52079287. Accessed 11 April 2020.

[CR40] Lashinsky, A. 2017. Tim Cook on How Apple Champions the Environment, Education, and Health Care. https://fortune.com/2017/09/11/apple-tim-cook-education-health-care/. Accessed 13 April 2020.

[CR41] Lanier J (2013). Who Owns the Future?.

[CR42] Latour B, Woolgar S (1979). Laboratory Life: The Construction of Scientific Facts.

[CR43] Lehtiniemi T, Ruckenstein M (2019). The social imaginaries of data activism. Big Data & Society.

[CR44] Lehtonen T, Liukko J (2010). Justifications for commodified security. Acta Sociologica.

[CR45] Love. S. 2016. Mark Zuckerberg and Priscilla Chan announce $3 billion effort aimed at curing disease. *STAT. *https://www.statnews.com/2016/09/21/mark-zuckerberg-and-priscilla-chan-announce-3-billion-effort-aimed-at-curing-disease/. Accessed 13 April 2020.

[CR47] Lupton D (2014). The commodification of patient opinion. Sociology of Health & Illness.

[CR48] McGoey L (2015). No Such Thing as a Free Gift: The Gates Foundation and the Price of Philanthropy.

[CR49] Moody M, Thévenot L, Lafaye C, Lamont M, Thévenot L (2000). Forms of valuing nature: Arguments and modes of justification in French and American environmental disputes. Rethinking Comparative Cultural Sociology: Repertoires of Evaluation in France and the United States.

[CR50] Mol A (2008). The Logic of Care: Health and the Problem of Patient Choice.

[CR51] Nozick R (1974). Anarchy, State and Utopia.

[CR52] Pascal, B. 1966. *Pensées*. Trans. A. J. Krailsheimer. Harmondsworth: Penguin.

[CR53] Pasquale F (2015). The Black Box Society: The Secret Algorithms that Control Money and Information.

[CR54] Powles J, Hodson H (2017). Google DeepMind and healthcare in an age of algorithms. Health and Technology.

[CR55] Powles J, Hodson H (2018). Response to DeepMind. Health and Technology.

[CR56] Prainsack B (2014). The powers of participatory medicine. PLoS Biology.

[CR57] Prainsack B (2017). Personalized Medicine: Empowered Patients in the 21st Century?.

[CR58] Prainsack B (2020). The political economy of digital data: Introduction to the special issue. Policy Studies.

[CR59] Radin M (1996). Contested Commodities.

[CR60] Rathenau Institute (2020). Datasolidariteit voor gezondheid (Data solidarity for health).

[CR61] Sandel M (2012). What Money Can’t Buy: The Moral Limitations of Markets.

[CR62] Satz D (2010). Why Some Things Should Not Be for Sale: The Moral Limits of Markets.

[CR63] Schayes, S. 2018. Why Amazon hiring a geriatrician is noteworthy. *MedCityNews*. https://medcitynews.com/2018/03/amazon-hiring-geriatrician-noteworthy/. Accessed 5 April 2018.

[CR64] Scheper-Hughes N (2000). The global traffic in human organs. Current Anthropology.

[CR65] Sharp LA (2000). The commodification of the body and its parts. Annual Review of Anthropology.

[CR6] Sharon T (2016). The Googlization of health research: From disruptive innovation to disruptive ethics. Personalized Medicine.

[CR7] Sharon T (2018). When digital health meets digital capitalism, how many common goods are at stake?. Big Data & Society.

[CR8] Sharon, T. 2020. Blind-sided by privacy? Digital contact tracing, the Apple/Google API and big tech’s newfound role as global health policy makers. *Ethics and Information Technology*. 10.1007/s10676-020-09547-x. 10.1007/s10676-020-09547-x PMC7368642 32837287

[CR66] Siffels L (2020). Beyond privacy vs. health: A justification analysis of the contact-tracing apps debate in the Netherlands. Ethics and Information Technology.

[CR68] Smith A (1776). An Inquiry into the Nature and Causes of the Wealth of Nations.

[CR69] Steinhubl S, Muse E, Topol E (2013). Can mobile health technologies transform health care?. JAMA.

[CR70] Sterckx S (2013). Trust is not something you can reclaim easily. Genetics in Medicine.

[CR24] The Economist. 2006. The birth of philanthrocapitalism. https://www.economist.com/special-report/2006/02/25/the-birth-of-philanthrocapitalism. Accessed 13 April 2020.

[CR71] Terranova T (2000). Free labor. Social Text.

[CR72] Titmuss R (1970). The Gift Relationship: From Human Blood to Social Policy.

[CR73] Topol E (2015). The Patient Will See You Now: The Future of Medicine is in Your Hands.

[CR74] Topol E (2019). Deep Medicine: How Artificial Intelligence Can Make Healthcare Human Again.

[CR75] Turakhia M (2019). Rationale and design of a large-scale, app-based study to identify cardiac arrhythmias using a smartwatch: The Apple Heart Study. American Heart Journal.

[CR76] Vaidhyanathan S (2011). The Googlization of Everything (and Why We Should Worry).

[CR77] Van Dijck J, Poell T, de Waal M (2019). The Platform Society: Public Values in a Connected World.

[CR78] Verbeek P (2005). What Things Do.

[CR79] Verily. 2019. Introducing OneFifteen. https://blog.verily.com/2019/02/introducing-onefifteen.html. Accessed 13 April 2020.

[CR80] Walzer M (1983). Spheres of Justice: A Defense of Pluralism and Equality.

[CR81] Walzer M (1984). Liberalism and the art of separation. Political Theory.

[CR82] Wilbanks J, Friend S (2016). First, design for data sharing. Nature Biotechnology.

[CR83] Ylä-Anttila T, Luhtakallio E (2016). Justifications analysis: Understanding moral evaluations in public debates. Sociological Research Online.

[CR84] Zelizer V (2011). Economic Lives: How Culture Shapes the Economy.

[CR85] Zuboff S (2019). The Age of Surveillance Capitalism: The Fight for a Human Future at the New Frontier of Power.

